# Thyroid Hormones Concentrations during the Mid-Dry Period: An Early Indicator of Fatty Liver in Holstein-Friesian Dairy Cows

**DOI:** 10.4061/2010/897602

**Published:** 2010-02-10

**Authors:** Horea Šamanc, Velibor Stojić, Danijela Kirovski, Milijan Jovanović, Horia Cernescu, Ivan Vujanac

**Affiliations:** ^1^Department of Farm Animal Diseases, Faculty of Veterinary Medicine, University of Belgrade, Bulevar Oslobodjenja 18, 11000 Belgrade, Serbia; ^2^Department of Physiology and Biochemistry, Faculty of Veterinary Medicine, University of Belgrade, Bulevar Oslobodjenja 18, 11000 Belgrade, Serbia; ^3^Department of Pathomorphology, Faculty of Veterinary Medicine, University of Belgrade, Bulevar Oslobodjenja 18, 11000 Belgrade, Serbia; ^4^Faculty of Veterinary Medicine, University of Timisoara, 1900 Timisoara, Romania

## Abstract

Relationship between postpartal fatty liver and thyroid gland activity during the peripartal and mid dry periods was studied. Twenty one dry cows were chosen. Blood samples were obtained on days −30, −2, and +12 related to calving and analized for thyroxine (T_4_) and triiodothyronine (T_3_). A T_3_/T_4_ ratio was calculated. Liver tissue samples were taken 12 d after calving and tested for the lipid content. Cows were divided into three groups: mild (<20% fat), moderate (20 to 30%), or severe fatty liver (>30%). Cows, that were affected with severe fatty liver, were hypothyroid prior to development of the condition due to lower T_4_ concentrations, and had significantly lower concentration of T_3_ and higher T_3_/T_4_ ratios than cows with mild and moderate fatty liver. Thus, hypothyroid state during mid-dry period may be an early indicator of postpartal fatty liver and may provoke T_3_/T_4_ ratio increase in this group of cows.

## 1. Introduction

Fatty liver is a major health problem in high yielding dairy cows in early lactation [1, 2]. Nutritional factors such as obesity are crucial in fatty liver etiology. Obese cows have a greater decrease in feed intake during peripartal period and, therefore, have a more severe negative energy balance during early lactation [3]. That leads to increased lipolysis of adipose tissue. Nonesterifed fatty acids (NEFA) that are released from the stored triacylglycerol (TAG) in the adipose tissue are readily taken up by the liver where they provide energy for liver function. If more NEFA arrive at the liver, that needed for energy purposes, the excess may be oxidized incompletely and generate ketone bodies [4] as well as converted to TAG for deposition. Under normal conditions, TAG are secreted from the liver as very-low-density lipoproteins (VLDL). Inadequate secretion of VLDL, however, contributes to development of fatty liver [5].

However, obesity in dry cows is not always associated with fatty liver indicating that there are risk factors other than obesity that predispose the cow to development of fatty liver [5]. Inadequate endocrine adjustments during initiation of lactation may play a role in fatty liver pathogenesis. In particular, the thyroid gland may play a role since the intensity of metabolic activity, metabolism of carbohydrates and lipids as well as lactogenesis are determined by hormones released from that tissue. There are evidences in literature about strong relation between thyroid hormone status during the peripartal period (3 weeks before to 3 weeks after calving), when the homeostatic mechanisms are under the greatest challenges, and postpartal metabolic disorders in Holstein-Friesian dairy cows [6, 7]. Although there is inconsistency in literature data due to experimental procedure, it is generally observed that thyroid hormone concentrations decrease during the peripartal period due to the adaptation of organisam to negative energy balance and initiation of lactation [8–12]. If reductions are pronounced or not synchronized with other metabolic and endocrine adjustments, the decrease may cause a metabolic disturbance that leads to increased lipolysis of adipose tissue and triglyceride accumulation in parenchymal tissue, especially the liver. Kapp et al. [13] observed that decreased levels of thyroid hormones in blood were associated with reduced mitochondrial capacity to oxidize fatty acids which led to diffuse lipid infiltration of hepatocytes and pronounce hepatic function impairment.

The hormonal status of cows in the far-off and mid dry period and its relationship to postpartal metabolic disorders, to our knowledge, have not been reported in the literature. With respect to fatty liver pathogenesis, identification of risk factors that are associated with homeorhetic mechanisms leading to metabolic disorders would provide a tool that could be used to better manage cows to prevent fatty liver or provide early warning signs that could be used to segregate cows and mark them for intensive interventional protocols after calving. This strategy requires an understanding of the point in time when adjustments of regulatory mechanisms break through physiological limits thus predisposing the cow to metabolic problems.

The aim of this study was to monitor thyroid gland activity from 30 days before expected calving to 12 days postpartum in order to investigate its relationship with liver lipid content on day 12 postpartum. The objective of the work was to develop a predictor of fatty liver that could be used for diagnostic and interventional purposes. 

## 2. Material and Methods

Twenty one healthy dry cows (30 days before expected calving) that had yielded 7000 liters of milk or more per annum in the previous lactation were chosen from the commercial dairy herd and placed on the study. The cows ranged from 4 to 6 years of age. The cows were housed in a tie-stall barn. Ingredients and chemical compositions of the dry and early lactation cow diets are listed in Tables 1 and 2.

Three blood samples were taken by jugular venipuncture from each animal: the first 30 days before expected calving (mid dry period), the second 2 days before expected calving (late dry period), and the third 12 days after calving (early lactation period). Samples were obtained with a sterile needle into tubes and allowed to clot spontaneously (approximately 15 minutes) on ice. Samples were subsequently centrifuged at 1,000 g for 20 minutes, and the serum was decanted and stored at −18°C until analyzed. To compare hormone concentrations without influence of daily rhythms, samples were taken 4 to 6 hours after morning feeding.

Triiodthyronine (T_3_) and thyroxine (T_4_) concentrations in the blood serum were measured by radioimmunoassay kit (INEP-Zemun, Serbia) validated for use with bovine serum. The mean intraassay coefficients of variation (CV) for duplicate samples were 4.1% and 3.5% for T_4_ and T_3_, respectively. Inter assay CVs were below 10%.

On day 12 after calving, liver percutaneous biopsies were obtained using a biopsy instrument following the method of Hojovcova and Kacafirek [14]. The biopsy was performed at the right 11th intercostal space, approximately 2 cm below the horizontal line through the tuber coxae. A medium-sized cannula (~6 mm o.d. and ~4 mm i.d; 20.5 cm long) surrounding a solid, retractable needle-pointed trocar was inserted through the muscle and peritoneum into the liver in the direction toward the left shoulder. After boring the cannula into the liver, a sample (3 to 5 cm long and 3 to 4 mm in diameter) was obtained through creating a vacuum by drawing back the trocar and then flexing the tip of the cannula upward and gently pushing forward. The liver sample was expelled onto a clean wipe, blotted free of blood, and placed into storage vials that contained 10% phormaldehyde solution for fixation.

Liver tissue lipid content was determined at the Pathomorphology Department at the Faculty of Veterinary Medicine, University of Belgrade. For pathohistological determination of lipids, sections were made using a freezing microtome and stained with Sudan III. Lipid content in the hepatocytes was determined through computer image analysis (Software Q Win).

Cows were divided into three groups of equal size based on the degree of lipid accumulation in the liver: mild fatty liver (<20% fat, *n* = 7), moderate fatty liver (20 to 30% fat, *n* = 7), and severe fatty liver (>30% fat, *n* = 7).

### 2.1. Statistical Analysis

Normality was tested using the Kolmogorov-Smirnov test before statistical analysis. As all variables showed normal distribution, statistical analysis was performed using two-way ANOVA, with time and the degree of lipid accumulation in the liver as fixed factors. Where ANOVA revealed a significant effect, Tukey *post hoc* test was administered to identify differences between groups. The Pearson correlation coefficients were also computed. All calculations were performed with SPSS 10.0 (SPSS Inc. Chicago, IL, USA). The differences were considered significant at *P* ≤ .05. All results are expressed as means  ± SD.

## 3. Results and Discussion

**Figure 1:**
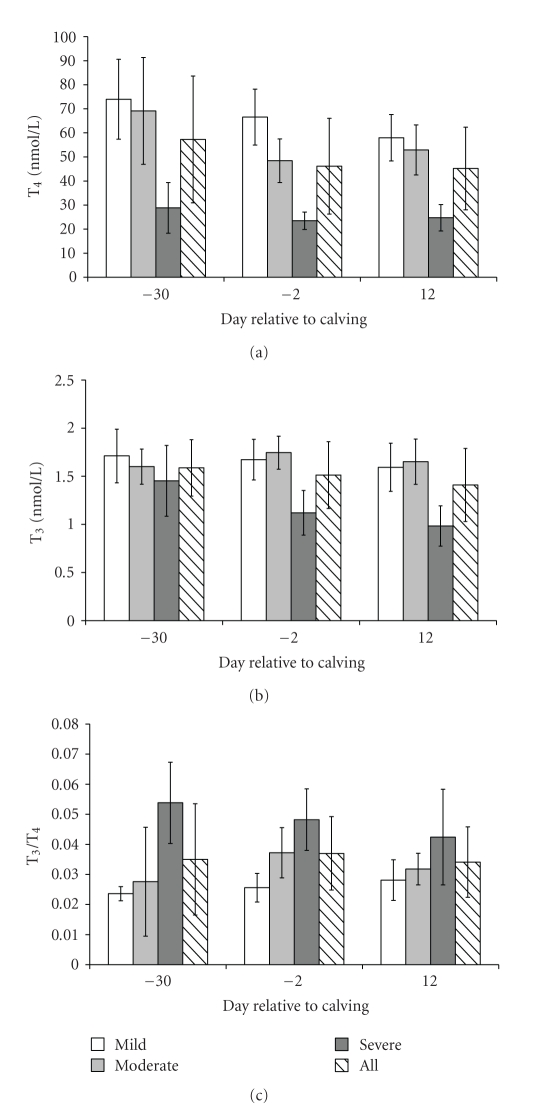
Serum levels (Mean  ± SD) of T_4_ in nmol/L (a), T_3_ in nmol/L (b) and T_3_/T_4_ ratio (c).

Analyzing data for all cows (diagonally patterned bar, Figure 1), T_4_ and T_3_ concentrations showed a downward trend from day −30 to day 12 after calving (Figures 1(a) and 1(b)), while the T_3_/T_4_ ratio increased from day −30 to day −2 and decreased from day −2 to day 12 (Figure 1(c)).

Our results are consistent with previous reports in the literature relative to the effects of time during the periparturient period on thyroid hormone concentrations in cows [8–12]. However, according to the results obtained by two-way ANOVA with time and the degree of lipid accumulation in the liver as the fixed factors, time was a signifant factor for T_4_ only (*F* = 6.34, *P* < .01, and *F* = 2.84, *P* = .067, *F* = 0.41, *P* = .667 for T_3_ and T_3_/T_4_ ratio, resp.). T_4_ concentration 30 days before expected calving significantly differed from values on 2 days before calving and 12 days of lactation (*P* < .05 and *P* < .01, resp.). The T_3_/T_4_ ratio is a simple calculated weight units ratio that reflect thyroid gland function and the potential for action of these hormones on peripheral tissues. The thyroid gland produces mainly T_4_, but also a small quantity of T_3_. In the peripheral tissues the mostly inactive T_4_ undergoes extrathyroidal enzymatic activation by 5′deiodinase (5′D) producing the much more potent T_3_ [15]. According to Pezzi et al. [12], type I 5′D prevails in liver and is most active during the dry period, while the highly efficient type II 5′D predominates in mammary glands, and its activity is the highest during early lactation.

According to content of lipids in liver at day 12 after calving, cows were divided into three groups: cows with mild fatty liver (*n* = 7; 10.34 ± 4.81% fat), cows with moderate fatty liver (*n* = 7; 25.77 ± 3.92% fat), and cows with severe fatty liver (*n* = 7; 43.58 ± 6.10% fat). Concentrations of T_3_, T_4_ as well as T_3_/T_4_ ratio were all dependent on the content of lipids in liver (*F* = 26.82, *F* = 63.42, and *F* = 24.12, resp., *P* < .001). Thus, T_4_ concentrations were significantly less for cows with severe fatty liver (Figure 1(a)) than for cows with mild (*P* < .001) and moderate (*P* < .001) fatty liver, and a slight but significant difference was also found between mild and moderate fatty liver (*P* < .05). Furthermore, cows with severe fatty liver had significantly lower concentration of T_3_ than cows with mild (*P* < .001) and moderate fatty liver (*P* < .001) (Figure 1(b)). The T_3_/T_4_ ratios (Figures 1(c)) for cows with severe fatty liver were significantly higher than for cows with mild (*P* < .001) and moderate fatty liver (*P* < .001). Interaction between time and the degree of liver lipids was insignificant for all parameters (*F* = 2.54, *P* = .055, *F* = 1.29, *P* = .287, and *F* = 1.64, *P* = .179, for T_3_, T_4_, and T_3_/T_4_ ratio, resp.).

Correlation coefficients between determined parameters (T_4_, T_3_, and T_3_/T_4_ ratio) and the content of lipids in the liver of cows are shown in Table 3.

This work demonstrates that dairy cows with severe fatty liver experience lower serum T_4_ concentrations before the peripartal period. Additionaly, the results of this study provide evidence of a significant negative correlation between T_4_ concentrations and the degree of fatty accumulation in the liver by 12 days after parturition not only during late-dry (*r* = −0.898; *P* < .001) and early lactation period (*r* = −0.769; *P* < .001), when lipid mobilization occurs, but also during mid dry period (*r* = −0.633; *P* < 0.01) (Table 3). Low thyroid hormone (T_3_ and T_4_) concentrations during the early lactation period in cows with severe fatty liver has already been established [8–11]. During the liver steatosis, fatty acids accumulate in the liver parenchyma, and it has been demonstrated that some fatty acids inhibit type-I liver 5′D activity [16]. That is probably the reason for low T_3_ concentrations during the late dry (day −2) and early lactation (day 12) periods in cows with severe fatty liver. Additionally, a significant negative correlation exists between T_3_ concentrations and the degree of fat accumulation in liver during the late dry (*r* = −0.680; *P* < .01) and early lactation (*r* = −0.606; *P* < .01) periods, but not during the mid dry period (Table 3). High T_3_/T_4_ ratios in cows with severe fatty liver during the mid dry period and a significant positive correlation with the degree of fat accumulation in liver (*r* = 0.606; *P* < .01) may indicate a pronounced decline of T_4_ levels or involvement of mechanism that increases T_3_ concentration. According to Pezzi et al. [12], T_3_ concentration increase could be due to increased 5′D tissue activity. Significant positive correlations between T_3_/T_4_ ratio and the degree of fat accumulation in liver exist until calving (*r* = 0.772; *P* < .001). Since liver is the main site of deiodinase activity during the dry period [12], it can be hypothesized that cows that will be affected with severe fatty liver had enhanced metabolic activity in the liver during mid dry period which may compromise hepatocyte adaptability during the peripartal period. Additionally, prevalence of cows with T_4_ concentrations below the lowest physiological value (4.2 *μ*g/dL, according to Kaneco et al. [17]), was significantly higher in cows with severe fatty liver during mid dry period than in cows with moderate and mild fatty liver (*P* < .01 both), while during late dry period the difference was statistically significant only between cows with severe and mild fatty liver (*P* < .01). Namely, all cows with severe fatty liver had T_4_ below cut off value, which indicates that they experienced hypothyroidsm before calving. Only one cow in group with moderate fatty liver and no cow in group with mild fatty liver had T_4_ concentrations below 4.2 *μ*g/dL.

In conclusion, we can affirm that cows, that were affected with severe fatty liver during early lactation, were in a hypothyroid state during the entire examining period, which means prior to development of the condition, too. The hypothyroid state during the mid dry period may be both a risk factor and an early indicator of fatty liver, while, during early lactation, it is important etiopathogenetic factor of fatty liver. According to our results, it may be suggested that low peripheral serum thyroid hormone status may provoke T_3_/T_4_ ratio increase. Based on the avaible data from this experiment, no conclusion related to mechanism that provokes T_3_/T_4_ ratio increase could be offered. In order to clarify that mechanism futher investigations are required.

## Figures and Tables

**Table 1:** Ingredients of cow's diets.

	Days related to calving		
Composition	−60 to −14 days	−14 to 0 days	0 to +60 days
Ingredient, kg			
Alfalfa hay	—	—	3.43
Grass hay	2.82	1.50	—
Wheat straw	1.80	0.60	—
Corn silage 44% DM	—	—	9.50
Corn silage 33% DM	10.0	10.0	
Corn silage 33.94% DM	—	—	9.00
Alfalfa haylage 51.79% DM	5.00	2.50	—
Alfalfa haylage 47.40% DM	—	—	5.00
Brewer's grain 21.00% DM	—	—	5.00
Corn grain	0.71	0.98	2.50
Barley grain	0.50	0.50	1.50
Soybean grits	—	0.30	1.30
Soybean meal 44% N	0.67	1.10	1.13
Wheat flour	0.50	0.50	1.30
Sugar beet pulp	—	—	1.82
DextroFat SC	—	0.10	0.40
Optigen II, 41% N	—	—	0.14
Dextrose monohydrate	—	0.04	0.10
Dicalcium phosphate 18% P	—	—	0.27
Magnesium oxide	—	—	0.05
Sodium bicarbonate	—	—	0.15
Sodium chloride (iodized)	—	—	0.07
Calcium carbonate	0.03	0.04	0.03
Milkinal trocken 3%	0.20	0.08	—
Beta carotene	—	0.004	—

Total	22.23	18.24	42.69

**Table 2:** Chemical composition of cow's diets.

	Days related to calving		
Chemical composition	−60 to −14 days	−14 to 0 days	0 to +60 days
DM kg	12.35	9.82	23.63
Net energy of lactation (NEL) MJ	72.86	65.50	163.03
Crude protein (CP) %	12.5	15.03	16.05
Rumen undegradable protein (RUP) %	4.09	5.09	5.06
Crude fat %	2.60	3.82	4.78
Acid detergent fibre (ADF) %	32.19	25.31	22.08
Neutral detergent fibre (NDF) %	49.53	40.59	35.48
Ca %	0.73	0.64	0.90
P %	0.44	0.42	0.52
Na %	0.23	0.13	0.36
Cl %	0.30	0.17	0.29
Mg %	0.31	0.27	0.34
K %	1.42	1.30	1.18
S %	0.23	0.22	0.22
Mn ppm	111.56	129.36	82.40
Cu ppm	33.23	39.81	25.64
Zn ppm	132.27	168.24	96.90
Co ppm	0.78	1.03	0.54
J ppm	2.44	3.06	1.64
Fe ppm	221.67	228.38	220.53
Se ppm	0.89	1.14	0.70
Vit A IU/kg	21 487.26	41 234.28	21 273.58
Vit D IU/kg	1 574.05	4 487.10	3 445.30
Vit E Iu/kg	83.31	117.79	69.35

**Table 3:** Correlation coefficients (*r*) between thyroid hormones in serum (T_4_, T_3_, and T_3_/T_4_ ratio) and the content of lipids in the liver 12 day after calving.

Parameter	30 days before expected calving	2 days before expected calving	12 days after calving
T_4_	−0.633**	−0.898***	−0.769***
T_3_	−0.334	−0.680**	−0.606**
T_3_/T_4_	0.606**	0.772***	0.492*

*P < .05; **P < .01, ***P < .001.
